# The dissemination effect of human-computer interactive advertising news—Using the theory of media audience and emotion management

**DOI:** 10.3389/fpsyg.2022.959732

**Published:** 2022-07-29

**Authors:** Zhu Xu, Chuanbin Zhang

**Affiliations:** ^1^Department of Humanities, Nanjing Normal University Zhongbei College, Zhenjiang, China; ^2^School of Journalism and Communication, Nanjing Normal University, Nanjing, China

**Keywords:** interactive advertising, news, advertising, media audience, emotion management, convolutional neural network, recognition

## Abstract

The development and application of network media has seriously impacted the social information dissemination environment dominated by traditional media. To break the dissemination barriers encountered by traditional media, this work probes into the dissemination effect of human-computer interactive advertising news. An in-depth analysis of the current dissemination situation of interactive online advertising (IOA) is firstly conducted, and then the methods to effectively guide and manage audience emotions are studied. Finally, an improved LeNet-5 model is established to identify audience emotions. The improvement of the LeNet-5 model in this work is composed of the following four points. (1) The convolution module sets Inception_conv3 and Inception_conv5 are adopted to replace the third convolutional layer Conv3 and the fifth layer Conv5 of the LeNet-5, respectively. (2) The size of the convolution kernel is changed. The original convolution kernel is replaced by two 3 × 3 convolution kernels in the Inception_conv3 and Inception_conv5 module sets. (3) The number of convolution kernels is reasonably changed. (4) The Batch Normalization (BN) layer is used. The experimental results show that interactive advertisements have the better dissemination effects among the audiences with older age, higher education, and in more developed cities. The improved LeNet-5 network can effectively solve the over-fitting and gradient disappearance, with a good robustness. The recognition rate reaches more than 81%, which is higher than the traditional LeNet-5 network by 3%. It can be known that the accuracy of the improved LeNet-5 network image recognition is significantly promoted. This research provides a certain reference for the optimization of news dissemination.

## Introduction

The development and application of online media has seriously impacted the social information dissemination environment dominated by traditional media. Interactive online advertising (IOA) generally refers to online advertising (OA) forms that rely on online media and can cause user behavior to communicate and interact. In terms of audiences for advertising dissemination, the base of advertising audiences is large, the age tends to be younger, and the occupations are mainly students and white-collar workers. Comprehensive portals, search engines, instant messaging, and websites are the types of websites they often come into contact with. Interactive banner advertising, rich media advertising, product advertising website, and search engine advertising have become several typical dissemination forms of IOA due to their strong technical support and friendly interactive content (Schmader and Horton, 2019; Sohn et al., 2019). In addition, IOA presents the advantages of diverse dissemination methods, diversified consumer activities, technical user use, good dissemination of information interaction, and good feedback channels. In the specific promotion process, although the interactive OA publicity integrates many advantages, it also faces many embarrassing situations in the actual promotion process, including the limited audience conditions, the vague scope of the target audience, the lack of consumer participation, single publicity channel, and low operability of content evaluation mechanism. The root causes of the above-mentioned difficulties in promotion can be attributed to the technical quality limitations of advertising agencies, the human factors limitations of advertisers, and the psychological factors of advertising consumers, and objective factors caused by constraints (Ajabshir, 2018). With the advent of the new media era, online media and mobile media characterized by interactivity have become new positions for advertisers to seize. From a macro perspective, the development of interactive advertising presents the mainstream characteristics of generalization of interactive carriers, complementary advantages of traditional media and new media, and difficult evaluation of interactive modes and effects. On the one hand, due to the rapid development of digitalization, various kinds of new media have been born, which has laid an excellent foundation for the development of interactive advertising. On the other hand, interactive advertising is new, and it is difficult to comprehensively and directly evaluate the coverage data such as the number of downloads, page views, and participants due to the fast update of the interactive carriers. The advertising, as one of the marketing methods, brings the actual effects on both the psychological level and the action level of consumers.

Many research works have been conducted in the corresponding field. Nicotra et al. (2018) studied interactive advertising starting with the concept of interactive advertising, and analyzed the current situation and characteristics of interactive advertising in the new media environment. Starting from the needs of the advertising industry, the author listed the problems of interactive advertising in the new media environment, and selected representative cases from the market for demonstration, which pointed out the direction for the promotion of interactive advertising (Nicotra et al., 2018). Meng and Huang (2022) empirically investigated how different advertising media independently and collectively affect a firm’s financial performance; The findings showed that both print and electronic media alone have a positive and significant effect on earnings, but each media form weakened the effectiveness of the other corresponding media (Meng and Huang, 2022). Kim et al. (2021) stated that the purpose of their study is to explore the impact of internet freedom, human rights, and economic freedom on socioeconomic progress in East Africa. It contributed to understanding the impact of predictors on social progress in specific areas of study. By investigating the impact of barriers such as Internet access, access to information and communication, personal freedom and choice, and economic freedom on the level of social progress index, it was found that these predictors had a significant positive impact on the socioeconomic level of the study area (Kim et al., 2021). Fondevila-Gascón et al. (2021) pointed out that past research has shown the important role of emotional appeal in charity advertising. Most research in this area has examined the effects of negative emotions, but when and how positive emotions are effective in encouraging donation allocation is less clear. The authors found through two experimental studies that congruent matching of pride with positive past performance and empathy with negative past performance increased donation allocation (Fondevila-Gascón et al., 2021). Ma (2021) pointed out that with the support of the fifth generation (5G) mobile communication technology, the development trend of China’s network information will become better and better. This work discusses the evolution and application of network convergence in the 5G era from a theoretical point of view, and proposes corresponding strategies. In the context of the rapid development of 5G technology, media convergence has become a trend. After expounding the advantages of attention mechanism in processing electroencephalo-graph (EEG) data under low-cost equipment and its own solution to the problem of memory deficit, the output value of the long-term and short-term memory network (LSTM) is separated from the hidden layer state value, and the designed attention module is introduced into it. Not only can the output value and hidden layer state value of the long-term memory network and the short-term memory network be maximized, but also good results are achieved, and more accurate data classification results are obtained (Ma, 2021). The main research flaw of the researchers is that there are many achievements in researching the effect of advertising on news from the commercial perspective in marketing and marketing. From the perspective of marketing, domestic researchers have discussed more about the management and social benefits of OA, but less about the impact of individual audiences and publicity effects.

The motivation of this work lies in that online video advertising has dual attributes of television and network. On the one hand, it can be a direct application of television advertising. On the other hand, it is rooted in the network platform and has a network interactive experience unmatched by television media. No matter from the point of profit or from the consideration of audience experience, the interactive online video advertising may become an important method to improve the effectiveness of online video advertising. At present, foreign researches about interactive online video advertising have achieved initial results, but domestic researches have just started and need to be further strengthened. The major innovation of this work is that the topic is relatively novel. The research of IOA has not yet mature. Exploratory research is carried out on the audience-centered IOA dissemination from its application. The issues and causes of IOA in the actual dissemination process are analyzed from the standpoints of advertisers and advertising agencies. This research has certain significance and value in both academic theory and practical operation. In academic theory, this work aims to provide a clear effect generation path for the dissemination mode of interactive advertising, so as to integrate advertising effect researches and contribute to the richness of interactive advertising researches.

## Interactive online video advertising and user experience

### The relationship between interactive design and user experience

The process of informatization has greatly changed the way of human existence, and it has also impacted the dominance of traditional network media. Due to the rapid development of network information technology, Internet advertising with the help of emerging information technology has become the main media platform for OA competition investment due to the advantages of diverse publicity channels, wide dissemination area, dissemination speed and timeliness, and two-way interaction (Yoon and Na, 2018; Wang, 2020; Ruvina, 2021). The amount of publicity OA is increasing year by year. In recent years, Internet media has entered the Internet age due to the emergence and development of virtual digital technology, which will also promote Internet advertising to have more diversified display methods and forms of publicity. At the same time, since the Internet age has strengthened the audience-centered propaganda concept of Internet advertising, a new Internet advertising model with the characteristics of the times, IOA propaganda, has emerged, and has created a new era of Internet advertising in China (Matsuda et al., 2021).

Interactive online advertising propaganda is not a prescriptive concept, but generally refers to the type of OA that is based on network media and can lead to exchange and interactivity of user behavior. In the industry, two technical indicators of hardware and software are generally used to measure the interactivity of OA, so as to judge the content of IOA. The so-called hard indicators, that is, formal technical indicators, generally include the following three aspects. The social environment generated by the online dissemination activity, the way of information content transmitted through the online dissemination activity, and the relationship between the participants of the online dissemination activity. Firsly, in the interactive OA activities, the relationship between the dissemination recipients and the recipients of online interactive advertisements composed of advertisers, advertising agencies, and audiences is equal, that is, the rights of publicity between the dissemination recipients and recipients are equal and influence can interact. Secondly, in the interactive OA activities, there are convenient, smooth, and effective information transmission channels and feedback channels between advertisers, advertising agencies, and target audiences. Thirdly, in interactive OA activities, advertisers, advertising agencies, and target audiences have the highest degree of virtual reality, with an immersive sense of presence. Soft indicators (content goals) refer to the publicity messages delivered in interactive online publicity campaigns, which should promote interaction between dissemination and recipients. This includes a key technical indicator, that is, the degree of personalization of publicity information. According to the degree of personalization of OA information content, the information content exchange between the sender and the receiver mainly presents the following three trends: cognitive interaction, emotional interaction, and friendship interaction in the interactive online advertising activities. The general content of OA information can lead to the general understanding of production and brand interaction among website audiences in the usual sense; and more personalized advertising information can establish emotional communication between dissemination and recipients. After careful market research, the personalized customized information content generated by precise positioning can provide an exclusive space between the dissemination person and the recipient, so that the recipient can have a deeper emotional exchange between the product and the brand.

**FIGURE 1:**
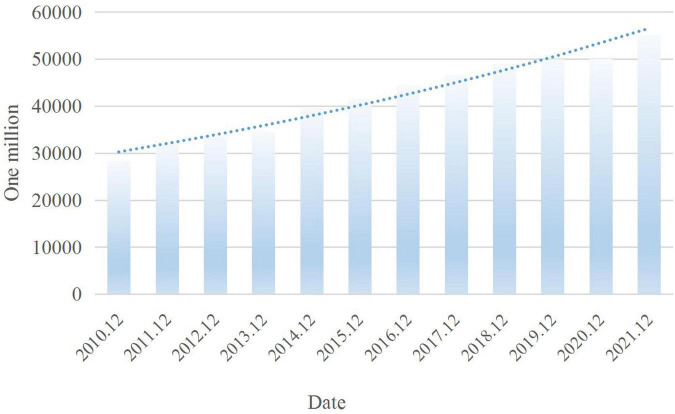
The changes in online video users.

**FIGURE 2:**
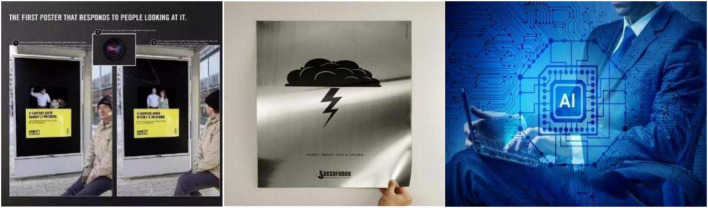
Representative interactive advertisements.

Figure 1 shows the changes in online video users from December 2010 to December 2021, and Figure 2 shows some representative interactive advertisements.

### Analysis of the issues and reasons in the dissemination of interactive online advertising

With the promotion and popularization of the Internet and intelligent terminals, IOA develops rapidly and becomes an important representative of new advertising. This is coupled with the wide spread of time and space, large information capacity, diverse forms of dissemination, accurate audience orientation, good audience experience, strong interaction between the senders and receivers, and more advantages of dissemination. The audience-centered dissemination of IOA not only stimulates audience participation to the greatest extent, but also enhances the dissemination effect of IOA, thus winning the favor of many advertisers. However, in practice, although audience-centered IOA dissemination has many advantages, it has encountered a series of embarrassing situations in the actual dissemination process. It has not performed well in attracting the attention of the audiences and further interactive participation, and the advertising effect is naturally not ideal. To improve the dissemination of audience-centered IOA, the existing issues in the dissemination process of IOA are analyzed from the advertisers, audiences, dissemination carriers, and advertising information on the basis of the analysis of the elements of the communication process. It can be divided into the following four points.

**FIGURE 3:**
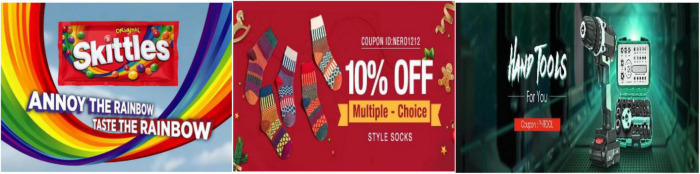
The common online advertisings (OAs).

(1) Audience attributes limit brand involvement. For its core content, it is hoped to establish some easy-to-remember, novel, and unique things for products in the competition period through specific advertising, so as to leave an appropriate psychological position of consumers. Every successful brand has a place in the minds of its audiences, and those audiences will develop into potential consumers of the brand. The audiences of audience-centered IOA tend to be younger, concentrated on students and white-collar workers, with the wide geographical distribution. That is, the audience-centered IOA already has a relatively clear audience population. Since the advertising dissemination is on the audiences, the existing audiences of IOA have a great initiative to choose the same or similar brand with their own philosophy and lifestyle. Therefore, those brands that do not target young audiences naturally do not need to choose this advertising method. From the above, the audience attributes of audience-centered IOA will limit the involvement of many brands (Zamith et al., 2020; Peiulis, 2021).

(2) The target audience is ambiguous. There are many situations in the actual dissemination process of audience-centered IOA. For example, advertisers or agencies will imagine their own dissemination objects out of thin air, or collect and analyze the information of dissemination objects still by traditional small-scale questionnaire surveys and online searches. Then one-sided survey results are sorted out according to these incomplete questionnaire data, so that the advertisers or advertising agencies’ cognition of the dissemination object is only superficial. There is no deep excavation of the deep correlation between the psychology, living habits, and more of the dissemination object and the product or brand. This phenomenon is especially prominent when new products or new brands enter the market. In this way, lightly, the contact points of dissemination will not fully cover the life trajectory of the communication targets, while in severe cases, the entire direction of the dissemination strategy will deviate from the dissemination target. Thereout, the dissemination effect will be impossible to gain.

(3) Insufficient audience participation. The starting point of audience-centered IOA is to maximize the enthusiasm of the audiences, so that the audiences can linger in the advertisement for a long time. Thus, the audiences can fully contact and experience the information about the product or brand in the advertisement. At this time, the participation of audiences brought by the integration of advertisements and products will transformed into use value. Audiences have also worked hard to participate in advertisements. If the audiences are interested in and like the products or brands in their advertisements, the audiences will further develop into potential consumers of the related advertisers’ products or brands. Then, the conversion of consumer value and the use value of advertiser selling goods is realized. The more deeply engaged the audiences is in the advertisement and the longer they linger, the more likely that price will convert. However, in the actual dissemination process, audience-centered IOA often encounter the insufficient audience participation. Specific performances include, the advertisement can’t attract the attention of the audiences, and the audiences quickly pull away after clicking into the advertisement page. Thereout, the dissemination strategy of the audience-centered IOA will become an empty talk only on paper, resulting in a great mass of waste of time cost, material cost, and labor cost of advertisers and advertising companies (Fang and Shi, 2019; Li et al., 2021).

(4) There is a single dissemination route. Smooth and rich media channels are the guarantee for the audience-centered IOA information to be smoothly conveyed to the target audiences. It is also a necessary condition to achieve the final advertising dissemination effect. However, in the actual dissemination process of the audience-centered IOA now, the terminal media that can carry the dissemination mainly focus on electronic products and the network, which is too single. In addition, advertisers and advertising companies have insufficient awareness of the audience-centered IOA media delivery strategy. Various online channels relying on the Internet have not been blooming, but concentrated on the portal sites with massive and comprehensive audiences and information and large-scale social networking sites with a high degree of comprehensiveness. This kind of rough media channel is too broad for the “scope of attack” of the audiences, so it is impossible to accurately target the target audiences. Figure 3 shows some common OAs.

## Research on media audience and emotion management program

### Audience sentiment analysis

**FIGURE 4:**
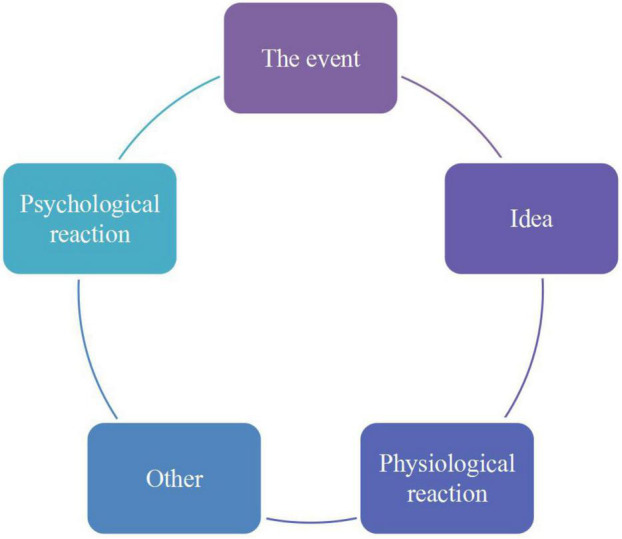
The source of emotions.

In advertising and news dissemination work, long-term research work has largely been based on discussions of techniques. The advertising and news dissemination work is not an isolated and one-way information dissemination, but needs to face the audience’s feedback on the advertising and news release work and combine the feedback to effectively carry out and improve the advertising and news dissemination work. In feedback research, the research on audience sentiment has become increasingly important in recent years. More and more cases have proved that audience sentiment is often affected by various factors, which in turn determine the audience’s acceptance of published content. From a psychological point of view, “emotional infection is the basis for establishing human interaction”; and from a dissemination point of view, information that can be accepted by the audience is effective information. Therefore, how to effectively influence and manage audience sentiment in publishing work is an important issue that current researchers must face (Kirchenbauer, 2020; Priyanto et al., 2021). Figure 4 shows the source of emotions.

### Research on emotion management

Emotion management work is divided in very different ways according to different criteria. According to whether the emotion can be consciously divided, it can be divided into conscious emotion management work and unconscious emotion management work. According to the different objects of emotion management, it can be divided into emotion management for self and emotion management for others. According to the different categories of management work, it can be divided into individual emotion management work and group emotion management (Mun et al., 2021; Park and Jin, 2021).

### Questionnaire design

Questionnaire survey is a commonly used scientific research method. The questionnaire survey method is adopted in this work by applying the computer technology to input the content of the questionnaire survey into the computer, and the investigator directly inputs the answer from the keyboard. The questionnaire in this work is designed with 20 questions. The questions are mainly multiple-choice questions, and there are a small number of rankings, which are not required to fill in the blanks. Investigators only need to make simple judgments of right and wrong opinions and judgments of whether they agree with attitudes and styles according to the questions. This work mainly investigates and analyses users’ awareness of interactive online videos, and analyses how different groups of people deal with various advertisements that appear in videos.

The questionnaire designed in this work is shown as Table 1.

**TABLE 1:** Questionnaire design.

Investigation of the interactive mode of online video advertising			
1. Have you seen video advertisements on the Internet?			
A. Yes	B. No		
2. When you see a web page embedded with a video advertisement, what kind of action do you generally take?			
A. Close the page immediately	B. Close the advertisement immediately	C. Watch part of the advertisement	D. Watch the advertisement entirely
3. When watching a video, how do you usually react to the advertisements that appear before and after the video?			
A. Watch the video after watching the advertisement	B. Silence the sound and wait	C. Do other things first, wait for the advertisement to finish before watching the video	D. Directly close the page without watching the advertisement or the video
4. When you watch a video and suddenly a floating advertisement appears on the periphery of the video, what kind of action do you usually take?			
A. Curious, take a few more glances	B. Curious, click to watch	C. Disgusted, close the advertisement	D. No feeling
5. When you are watching a video, how do you usually do to the background image advertisement outside the video?			
A. Never pay attention	B. See only, don’t click	C. Click occasionally	D. Click often
6. When you watch a video advertisement, what kind of action do you generally take?			
A. Watch partially	B. Watch in full	C. Watch in full and willing to share	D. Watch in full, share, and actively participate in discussions

### Application of consumer sentiment in interactive advertising design

In recent years, due to the emergence of various products and commodities, the market competitiveness of all walks of life is constantly increasing. Advertising is an effective means to enhance market competitiveness. Therefore, enterprises must pay more attention to advertising art design, incorporate emotional factors into the process of advertising art design. Thereby, consumers can notice the product and have a strong interest in the product. Only by attracting the attention of consumers first, can consumers pay attention to and understand the basic information and connotations generated, and finally promote the purchasing behavior. In advertising art design, adding human-specific emotional factors allows to correctly grasp the psychology of consumers, and can transform the basic information of commodities into culture and art to a certain extent. The following principles should be followed when applying emotional factors to advertising art design. First of all, the integration of emotional factors into the advertising art design should be able to attract the attention of consumers. Thus, consumers have the desire to know more about the product. If the emotional factor is added but it can’t attract the attention of consumers, then the follow-up work will not be carried out, and it will not play a role in promoting the consumption of the product. Therefore, when integrating emotional factors, the most basic thing is to attract the attention of consumers. Secondly, different emotional incentives should be incorporated for different groups of people when incorporating emotional factors. Different consumer groups have different emotional incentives that can promote their consumption. For example, for women, if more aesthetic things are integrated into the advertising art design, it can bring visual enjoyment to consumers, thereby promoting female consumption. For male consumers, too much aesthetic experience may not induce their consumption, but only by emphasizing the practicality of the product can call their consumption. Therefore, the integration of emotional factors into advertising design should be considered for the consumer groups, and emotional incentives that match the consumer groups should be added. Finally, for integrating emotional factors into advertising art design, blindly dissemination of product information using emotional offensive is not inadvisable to achieve the purpose of selling products. This will not only fail to promote consumption, but will make consumers disgusted and have a bad impression of the product. Therefore, in advertising art design, incorporating emotional factors should start from the rational needs of consumers. With rational needs as the main requirement, some emotional motives can be appropriately added in design, which can effectively promote consumer consumption.

There are a large number of audiences when traditional advertisements are played. In addition, advertisers also need to control the time of advertisements, so it is difficult to consider the emotional state of each audience and make corresponding adjustments, which affect the spread of advertisements. Therefore, it has become an urgent issue to be solved currently to timely and effectively feed the emotional state of the audiences when watching the advertisements back to the advertisers.

## Audience emotion recognition based on convolutional neural network

### Model design

**FIGURE 5:**
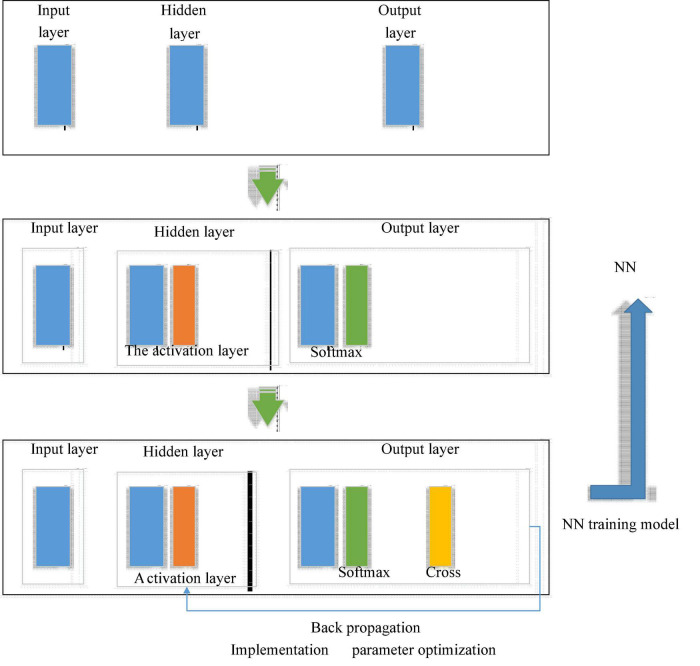
The structure of convolutional neural network (CNN).

The most critical technology for facial expression recognition lies in feature extraction, many methods are cumbersome to extract, and convolutional neural network (CNN) is a special multi-layer perceptron. At present, many convolutional structures used in facial expression recognition are complex, with many parameters and a large amount of computation; and the LeNet-5 algorithm can be directly used in facial expression recognition. However, the recognition rate of this algorithm is low, and the improved LeNet-5 algorithm after introducing the shallow convolution structure can extract richer detailed features. The recognition rate has been improved on the Japanses Female Facial Expression Database (JAFFE) database. CNN is a kind of feedforward neural network with convolution calculation and deep structure proposed in the 1990s, and it is one of the representative algorithms of deep learning. Figure 5 shows a structure diagram of simple CNN (Tumbas et al., 2018; Mrkajic et al., 2019; Kelebek and Alniacik, 2022).

The LeNet-5 network model was proposed by Yann LeCun, the father of CNN in 1998, and was the origin of a large number of neural network architectures in the past 20 years (Brown et al., 2018). The LeNet-5 network model uses convolution, parameter sharing, down-sampling, and other operations to extract features, and uses fully connected neural networks for classification and recognition, avoiding a lot of computational costs. The traditional LeNet-5 network model is improved in this work. The improvements in this work can be divided into the following aspects: (1) the BN layer is replaced with Z-score normalization, and (2) the Sigmoid function is substituted with the Relu activation function. Aiming at the issues of the traditional LeNet5 network in identifying user emotions, this work makes the following improvements. (1) The convolution module sets Inception_conv3 and Inception_conv5 are utilized to replace the third convolutional layer Conv3 and fifth layer Conv5 of the LeNet-5 network, respectively. The network depth is increased while more rich features of the target can be extracted. The two convolutional module sets contain 2-3 convolutional layers, respectively. (2) The selection of the convolution kernel is related to whether effective features can be extracted. The original 5 × 5 convolution kernel is not effective for traffic sign feature extraction. Therefore, the size of the convolution kernel is changed. In Inception_conv3 and Inception_conv5 module sets, the original convolution kernel is replaced by two 3 × 3 convolution kernels. (3) The traditional LeNet-5 network has a small number of convolution kernels in each layer, which can’t fully extract the rich features of the target. Thus, the number of convolution kernels is reasonably changed. (4) The Batch Normalization (BN) layer is utilized to normalize the input batch samples, so as to improve the input of the neural network, which can improve the network training speed to a certain extent.

**FIGURE 6:**
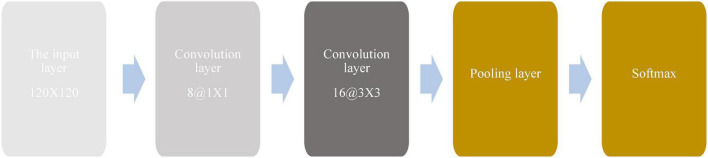
Improved LeNet-5 network model.

Figure 6 shows the improved LeNet-5 network model.

The training processs of the improved network can be divided into 7 steps:

(1) It should initialize the network. It refers to determining the parameters of the network according to the input sequence of the network (for example, the number of nodes in the input layer is n, the number of nodes in the hidden layer is l, and the number of nodes in the output layer is m), initializing the link weights ω_*i*,*j*_ and ω_*j*,*k*_ between neurons in each network layer, and finally giving the learning rate and activation function.

(2) It should calculate the output of hidden layer *H*_*i*_.


(1) $$H_{i}=f\,\left(\sum_{i=1}^{n}\omega_{i\,j}\,x_{i}-a_{j}\right)\,j=1,2,\ldots ,l$$

In the above Eq. 1, *i* is the number of hidden nodes, *f* is the hidden layer excitation function, *a*_*j*_ is the hidden layer threshold, and ω_*ij*_*x*_*i*_ is the neural unit link weight.

(3) It should calculate the predicted output *O*_*K*_ of the BP network.


(2) $$O_{K}=\sum_{j=1}^{l}H_{j\,\omega_{j\,k}}-b_{k}$$

(4) It should calculate the predicted error *e*_*k*_ of the network.


(3) $$e_{k}=Y_{k}-O_{K}$$

*Y*_*k*_ is the input of the neural network.

(5) It should update the link weights of neurons in the network according to the network prediction error *b*.


(4) $$\omega_{i\,j}=\omega_{i\,j}+\eta \,H_{j}\,\left(1-H_{j}\right)\,x\,\left(i\right)\,\sum_{k=1}^{m}\omega_{j\,k}\,e_{k}$$

(5) $$\omega_{j\,k}=\omega_{j\,k}\,\eta \,H_{j}\,e_{k}$$

(6) The node thresholds *a* and *b* are updated according to the network prediction error *e*.


(6) $$a_{j}=a_{j}+\eta \,H_{j}\,\left(1-H_{j}\right)\,\sum_{k=1}^{m}\omega_{j\,k}\,e_{k}$$

(7) $$b=b_{k}+e_{k}$$

(7) It can determine whether the algorithm iteration is over. If not, it has to return to the step 2).

The decoding equation can be expressed in the form shown in Eq. 8:


(8) $$F\,\left(b_{i\,l},b_{i\,2},b_{i\,l}\right)=R_{i}+\frac{T_{i}-R_{i}}{2^{l}-1}\,\sum_{j\,1}^{l}b_{i\,j}\,2^{j-1}$$

(*b*_*i*1_,*b*_*i*2_,…*b*_*il*_) in Eq. 8 is the i-th segment of each individual whose segment length is 1, and the meanings of *T_i_* and *R_i_* are the left and right endpoints of the definition domain of the segment component.

### Experimental configuration

Model is trained by using the graphic processing unit (GPU) server. The hardware configuration is Intel E52665X2, 32 GRECC DDR3, 250G SSD, and 4 NVIDIA RTX 2080TI 11G graphics cards; and the software configuration is Ubuntu Linux 16.04, CUDA10.0, cuDNN7.6. The test is performed on a laptop. The hardware configuration is Intel i79750H 4.5 GHz 6 cores, memory 32 G DDR4 2666, GPU is GeForce GTX 1650; and the software configuration is Windows10, CUDA10.1, Cudnn7.6, OpenCV3.4.1 (Schwarz, 2021; Ozanne et al., 2022).

In this work, the recognition rate and recognition time are used to assess the performance of the model. The calculation of the recognition accuracy can be expressed in the form shown in Eq. 9.


(9) Accuracy=Correctpredicted⁢number/Projectedtotal

## Analysis of experimental results

### The reliability and validity of the questionnaire

**FIGURE 7:**
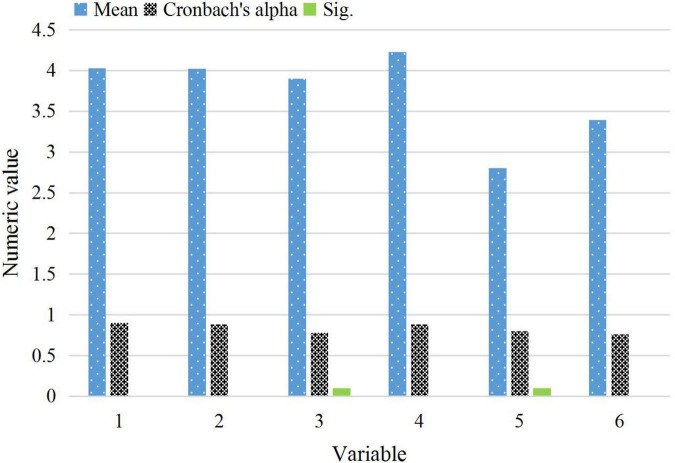
Cronbach coefficient and Sig value for reliability test.

This survey was conducted from February 9th to February 15th, 2021. It was distributed randomly on the Internet and forwarded through the Internet. 252 valid questionnaires are collected in the survey in total. According to the basic personal information filled in by the respondents, the following outcomes are obtained. The male to female ratio of the respondents is about 9:13, and women pay more attention to online video advertising than men. The respondents can be divided into four age groups: ≤17, 18–25, 26–40, and >40 years old. The age of the respondents is mainly concentrated in 18–25 and 26–40 years old, accounting for 49.78% and 45.78% of the total number of respondents, respectively. These are the population who pay the most attention to online video advertising, with relatively broad attitudes. This provides a strong support for the conclusion of this work. The reliability of each measurement item in the questionnaire is tested. The Cronbach coefficient and Sig value of the reliability test are shown in Figure 7.

As illustrated in Figure 7, the designed questionnaire is more reasonable and reliable, and meets the reliability test standards. The Cronbach coefficient of the designed questionnaire is 0.922, and the significance index (Sig) value is 0.000. The test results show that the internal measurement items have good internal consistency, reliability, and repeatability. The questionnaire is reliable.

**FIGURE 8:**
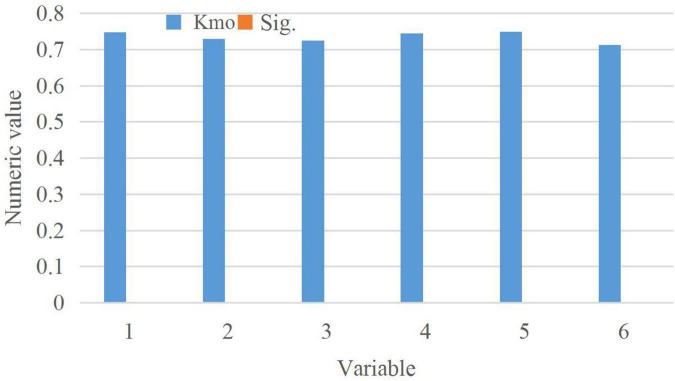
Statistical results of Kaiser-Meyer-Olkin (KMO) value and Bartlett’s spherical test.

The Kaiser-Meyer-Olkin (KMO) value of each variable in the questionnaire and the statistical results of Bartlett’s spherical test are shown in Figure 8.

Figure 8 demonstrates that the KMO test value of each variable in the questionnaire is greater than 0.7, all Sig values are 0, and the spherical test chi-square value is greater than 200. This shows that the variables in the questionnaire are suitable for factor analysis.

### Questionnaire results

**FIGURE 9:**
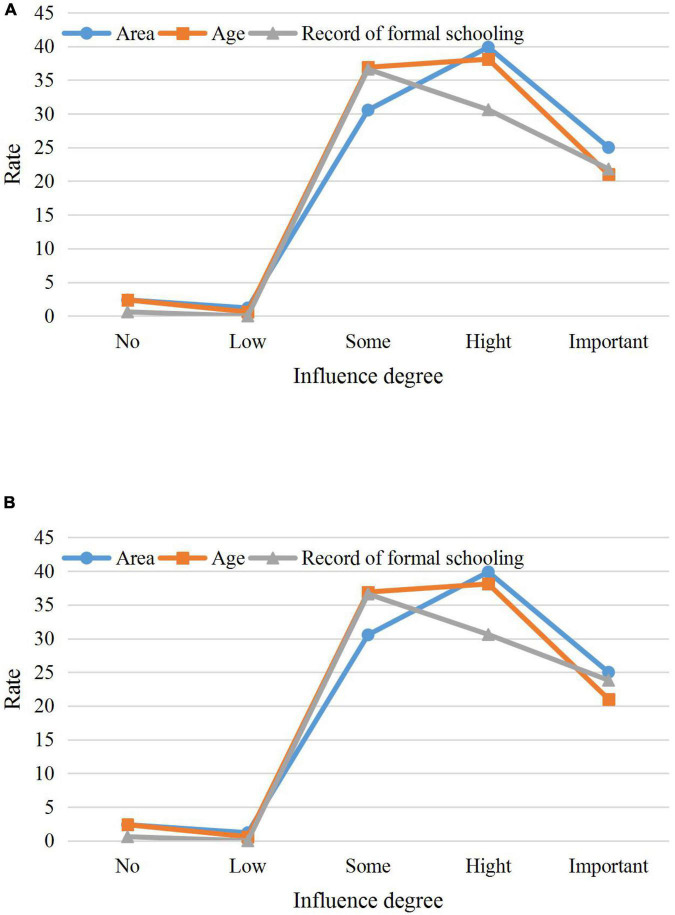
The questionnaire results. **(A)** The first test result; **(B)** The second test result.

Figure 9 shows part of the questionnaire results and reveals that the educational level of the respondents has the least influence on the processing method of embedded advertisements in web pages. The individual’s liking of various video advertisements basically shows a positive correlation. The older the surveyed age, the higher the education level, and the more developed the city they are in, the more obvious the way they understand video advertising.

### Experiment results based on deep learning

Table 2 shows the recognition results of the improved CNN on audience expressions.

**TABLE 2:** The recognition results of audience expressions.

Size of convolution kernel		Recognition rate (%)	Time (s)
1 × 1 × 8	3 × 3 × 16	81.4	15.1
3 × 3 × 8	5 × 5 × 16	65.12	22.1
5 × 5 × 8	7 × 7 × 16	76.14	33.2
7 × 7 × 8	9 × 9 × 16	76.14	46.1
9 × 9 × 8	11 × 11 × 16	76.14	55.2

As displayed in Table 2, 8 1 × 1 and 16 3 × 3 convolutional structures show the recognition rate to 81.40%; 8 1 × 1 double the input data to solve the problem of less data; and 16 3 × 3 convolution kernels show rich extracted features, with high recognition rate, reduced parameters, and fast convergence.

## Conclusion

With the new changes in the network environment, media pattern, audiences, etc., new forms of OA are derived. The current situation of IOA dissemination is first introduced. On the basis of analyzing the current situation of dissemination, the characteristics of audience-centered IOA dissemination are summarized from the aspects of advertising dissemination object, dissemination method, advertising audiences, transmission and reception parties, feedback channels, etc. Then, from the five elements of dissemination, the issues of audience-centered IOA and the subjective and objective reasons behind these issues are analyzed. The conclusions are drawn as follows. In the process of audience-centered IOA, the advertisers’ products or brands tend to be nationalized, fashionable, and younger. For advertising media, mobile network media applications rise ferociously. Advertising audiences tend to be younger, which are mainly students and white-collar workers. The audience-centered IOA dissemination presents the characteristics of audience differentiation, terminal operation technicalization, two-way interaction between transmission and reception, and highly effective feedback channels. Audience-centered IOA encounters issues such as audience attributes limiting brand involvement, blurred target audience, insufficient audience participation, single dissemination route, and poor flexibility of the effect evaluation system. That’s because of the subjective reasons arising from human factors of advertisers, advertising agencies, and advertising audiences, and objective reasons such as technical limitations and dissemination platform limitations. In the actual dissemination process of audience-centered IOA, all efforts should be made to clarify brand positioning, identify target audiences, improve advertising performance, expand media channels, and strengthen effect evaluation. Due to the limited ability of the authors, this work still has obvious shortcomings, such as the lack of comprehensive, convincing, and representative data when the current situation of audience-centered IOA is analyzed. The deficiencies in the research need to be further studied in the follow-up work.

## Author contributions

Both authors listed have made a substantial, direct and intellectual contribution to the work, and approved it for publication.
